# Cuproptosis- and m6A-Related lncRNAs for Prognosis of Hepatocellular Carcinoma

**DOI:** 10.3390/biology12081101

**Published:** 2023-08-08

**Authors:** Yuezhi Zhu, Jen Kit Tan, Jo Aan Goon

**Affiliations:** Department of Biochemistry, Faculty of Medicine, Universiti Kebangsaan Malaysia, Kuala Lumpur 56000, Malaysia

**Keywords:** hepatocellular carcinoma, cuproptosis, N6-methyladenosine, RNA methylation, LncRNAs, prognosis

## Abstract

**Simple Summary:**

Hepatocellular carcinoma (HCC) is known for its poor prognosis, but the markers for prognosis still remain unclear to date. This study focused on the long non-coding RNAs (lncRNAs) that linked with cuproptosis and N6-methyladenosine (m6A) in HCC, using data from the The Cancer Genetic Atlas (TCGA) database. The identified lncRNAs were used to develop a risk assessment model through specific types of statistical analysis. This model helped explore how different risk profiles affect the tumor’s genetic mutation load and the surrounding immune environment. Five particular lncRNAs were found to be significant in survival rates, and certain genes (TP53 and CTNNB1) were frequently mutated in high-risk patients. Interestingly, high-risk patients with a low genetic mutation load had the poorest survival rates, while low-risk patients with a high mutation load had the best survival rates. Furthermore, high-risk cases showed increased activity in certain cellular pathways, such as the cell cycle and glucose production. This study resulted in a promising lncRNA model that could potentially help in assessing and managing HCC. However, further studies are needed to confirm these findings.

**Abstract:**

Cuproptosis and N6-methyladenosine (m6A) have potential as prognostic predictors in cancer patients, but their roles in hepatocellular carcinoma (HCC) are unclear. This study aimed to screen a total of 375 HCC samples were retrieved from the TCGA database, and lncRNAs related to cuproptosis and m6A were obtained through correlation analysis. To construct a risk assessment model, univariate Cox regression analysis and LASSO Cox regression were employed. Analyze the regulatory effect of relevant risk assessment models on tumor mutation load (TMB) and immune microenvironment. A total of five lncRNAs (AC007405.3, AL031985.3, TMCC1-AS1, MIR210HG, TMEM220-AS1) with independent overall survival-related risk models were obtained by LASSO survival regression. TP53 and CTNNB1 were the three genes found to have the most mutations in high-risk group patients. The high-risk group with low TMB had the worst survival, whereas the low-risk group with high TMB had the best survival. KEGG pathway analysis revealed that the high-risk group was enriched with cell cycle, oocyte meiosis, cell senescence, and glycolysis/glucose production pathways. We constructed a reliable cuproptosis- and m6A-related lncRNA model for the prognosis of HCC. The model may provide new insights into managing HCC patients, but further research is needed to validate it.

## 1. Introduction

Hepatocellular carcinoma (HCC) is a pervasive primary liver tumor worldwide, characterized by a significant mortality rate [1]. The disease is known for its insidious onset, rapid growth, robust invasiveness, and high incidence [2]. Therefore, gaining a better understanding of the molecular mechanisms underlying HCC is crucial for developing new diagnostic methods and identifying potential therapeutic targets [3]. Current studies have shown that HCC ranks fourth in morbidity and second in mortality among malignant tumors in China [4,5,6]. Because its early stage is not easy to detect due to the absence of typical symptoms and signs, many patients cannot get early diagnosis and treatment [4,5,6]. Therefore, diagnosis is often accompanied by tumor metastasis and complications with poor prognosis. The pathogenesis of HCC is complex, and its etiology and molecular mechanism have not been fully understood. Epidemiological data showed that alcoholic fatty liver disease and non-alcoholic steatohepatitis are related to the incidence of HCC [7]. In addition, the prevalence of non-alcoholic steatohepatitis is increasing every year in China, which further develops into liver cirrhosis and HCC. Although liver resection and local ablation can cure patients with early-stage liver cancer, the high recurrence and metastasis rate of primary liver cancer limit the long-term survival of patients [8]. Therefore, there is an urgent need to construct an early prediction model of HCC and find reliable targets for targeted therapy.

Copper is an essential trace element that plays a crucial role in mitochondrial respiration and antioxidant/detoxification processes. Studies have found that the levels of copper in serum and tumor tissues of patients with cancer (including thyroid, gastric, and prostate cancer) are significantly altered, and copper is closely related to cancer etiology and progression [9]. For example, Wilson’s disease (disorder with abnormal copper accumulation) has been linked to an increased incidence of liver cancer, and copper levels are associated with the development and progression of pancreatic and prostate cancer [10]. Therefore, various types of copper ionic carriers, such as dithiocarbamate, thiosemicarbazone ligand, 8-hydroxyquinoline, and flavonoids, have been used in anticancer therapy [11,12]. In 2022, Tsvetkov et al. systematically reported for the first time the mode of cell death known as “cuproptosis”, which is mediated by mitochondrial-dependent respiration, lipoacylation of proteins, programmed necrosis, autophagy, and death [13]. Since then, more research on novel copper-binding compounds has been conducted to improve the precision and selectivity of anticancer therapy by targeting copper levels. In recent years, researchers have paid attention to the role of cuproptosis-related genes in HCC survival prediction, immune cell infiltration, and diagnostic value [14,15,16,17,18,19,20]. A large body of evidence suggests that cuproptosis-related genes have significant development value in HCC diagnostic applications [14,15,16,17,18,19,20]. However, the deeper mechanisms involved in these studies are still lacking, which is one of our points of interest.

In recent years, RNA methylation has become one of the focus points of epigenetics research due to the rapid development of people’s interest and understanding of RNA modification [21]. RNA methylation modifies purine and pyrimidine’s exocyclic nitrogen as well as carbon atoms and oxygen atoms of the 2′-OH part. This process includes N6 methyladenosine (m6A), 5-methylcytosine (m5C), N7-methylguanosine (m7G), 2′-O methylation (2′-o-Nm), and N1-methyladenosine (m1A) [22]. It is worth noting that RNA methylation is a dynamic and reversible process, and enzymes and downstream factors related to RNA methylation modification may be targetable to regulate the level of RNA methylation. This regulation could, in turn, affect RNA processing and metabolism, leading to modulation of cell proliferation and migration, resulting in physiological or pathological changes [22]. m6A is a dynamic modification throughout all processes of RNA metabolism, catalyzed by a methyltransferase (known as the writer) or reduced by demethylase or erasers, and recognized by m6A-binding proteins (reader) for its modification. Current studies have confirmed that m6A modification can regulate mRNA expression to exert biological effects. In addition, a large number of studies have confirmed the important role of m6A in the regulation of HCC pathogenesis [21,23]. However, few studies have focused on the role of RNA methylation in cuproptosis and its relationship with HCC.

Non-coding transcripts exceeding 200 nucleotides are knowns as long non-coding RNAs (lncRNAs) [24]. Research has demonstrated that lncRNAs act as either oncogenes or tumor suppressors and play a role in the regulatory processes of cancer initiation and progression [25]. Along with modulating the proliferation and differentiation of cancer cells, lncRNAs also regulate the metabolic reprogramming of these cells and are implicated in cancer cell invasion and metastasis [26]. Currently, several studies have reported the predictive role of m6A-related lncRNAs in HCC. For instance, Chen et al. identified 61 m6A-associated lncRNAs and two HCC subtypes with different clinical characteristics, as defined by consistent clustering of m6A-associated lncRNAs. The study found T significant differences in progression-free survival (PFS), expression of three tumor microenvironment (TME)-related scores, 15 immune-related gene sets, and two immune checkpoints between the two subtypes [27]. Another study by Hao et al. highlighted the potential prognostic value of m6A-associated lncRNAs in HCC by reporting the significant differences in the expression levels of m6A-lncRNAs in HCC [28]. However, more precise prognostic models are necessary for the precise treatment of HCC. Therefore, combining additional biomarkers to construct more reliable prognostic models is urgently needed. In this study, we aimed to develop mcplncRNA models by integrating m6A regulators and cuproptosis-related genes with specific lncRNA pairs, which may enhance the accuracy of HCC prognostic models.

## 2. Materials and Methods

### 2.1. Datasets Retrieval

We obtained RNA-Seq data for gene expression and accompanying clinical and mutation data for Liver Hepatocellular Carcinoma (LIHC) from the Cancer Genetic Atlas (TCGA) database (https://portal.gdc.cancer.gov, accessed on 2 February 2023). Additionally, we calculated the tumor mutation load (TMB) using Perl software by downloading tumor mutation data from the same database.

### 2.2. Identification of Cuproptosis-Related lncRNAs

To distinguish lncRNAs according to the gene annotation in TCGA, we obtained expression matrices of lncRNAs and cuproptosis genes in the TCGA database. Based on previous studies, we identified and retrieved 19 cuproptosis genes in TCGA (ATP7A, ATP7B, CDKN2A, DBT, DLAT, DLD, DLST, FDX1, GCSH, GLS, LIAS, LIPT1, LIPT2, MTF1, NFE2L2, NLRP3, PDHA1, PDHB, SLC31A1).

We performed a co-expression correlation analysis of lncRNAs and cuproptosis-related gene expression profiles using R packages, including limma, ggalluvial, and “ggplot. We plotted a Sankey diagram to visualize the results. One-way survival analysis was conducted using the Survival and survminer packages in R, with a significance level of *p* < 0.001 and a correlation threshold of |R| > 0.4. The results were visualized using forest plots, with significance set at *p* < 0.05.

We defined lncRNAs associated with cuproptosis using relevant standards and identified 23 lncRNAs significantly correlated with at least one cuproptosis-related gene (|Pearson r| ≥ 0.3, *p* < 0.05). These biostatistical methods provide valuable insights into the relationship between lncRNAs and cuproptosis in HCC and have potential applications for developing novel diagnostic and therapeutic strategies.

### 2.3. Identify the m6A-Related lncRNAs

RNA m6A methyltransferase is a complex of writer proteins, including METTL3, METTL14, METTL16, RBM15, RBM15B, VIRMA, WTAP, and ZC3H13. This gene is responsible for the m6A modification of RNA, which can be removed by m6A demethylase (eraser) enzymes, such as FTO and ALKNH5. This dynamic process of m6A modification and de-modification plays an important regulatory role in gene expression, and dysregulation of this process has been implicated in the pathogenesis of various diseases. The m6A modification mainly affects the fate of mRNA through its recruited reader proteins, including FMR1, HNRNPA2B1, HNRNPC, IGFBP1, IGFBP2, IGFBP3, LRPPRC, YTHDC1, YTHDC2, YTHDF1, YTHDF2, and YTHDF3. We used m6A-relevant standards to define lncRNAs and identified 23 lncRNAs significantly related to the expression of at least one m6A gene (|Pearson R| ≥ 0.3, *p* < 0.05).

### 2.4. Construction of m6A and Cuproptosis-Related lncRNA Mortality Risk Model for HCC

The TCGA-HCC dataset was randomly divided into training and test sets at a 1:1 ratio. In the training set, we developed a cuproptosis-associated lncRNA model using the least absolute shrinkage and selection operator (LASSO)-Cox regression analysis, and we validated the model using the test set. We conducted univariate Cox analysis in the training set, followed by LASSO-Cox regression analysis to identify lncRNAs associated with cuproptosis and m6A. We used 1000-fold cross-validation of significantly expressed lncRNAs for this purpose. We identified the best prognostic lncRNA using multivariate Cox regression analysis with a significance level of *p* < 0.05. We used the best model parameters for feature construction and risk score calculation. For LASSO-Cox regression analysis, we used the glmnet function in R with 10-fold cross-validation of estimated penalty parameters. We developed the lncRNA risk model associated with cuproptosis and m6A and the risk score using the formula:

coef (lncRNA1) ∗ expr (lncRNA1) + coef (lncRNA2) ∗ expr (lncRNA2) + ... + coef (lncRNA n) ∗ expr (lncRNA n)

We used the R packages for heat map, survival, and survminer to divide data into high-risk and low-risk groups based on the risk scores, survival status, survival time, survival curves, and lncRNA expression heat map in the model.

### 2.5. Evaluation of the Grouping Ability of Risk Scores

We used R packages survminer and survivor to assess the difference in overall survival (OS) between high- and low-risk groups with different clinical characteristics by employing Kaplan–Meier survival analysis. Additionally, we conducted principal component analysis (PCA) on the distribution of patient data with different risk scores using limma and scatterplot3D within the R package.

### 2.6. Evaluation of the Validity of the Model

In order to evaluate the independent prognostic values of risk prediction features, the survival package in R was used to determine OS and PFS in various groups. Additionally, the survival ROC package was utilized to calculate the area under the curve (AUC) values for 1-year, 3-year, and 5-year intervals of features under receiver operating characteristic (ROC) curves in the training, testing, and all groups. Moreover, to plot age, grading, and risk scores and illustrate the deviation between predicted and actual results, a calibration curve was generated using the rms and pec packages in R, which was further used for consistency index (C-index) calculations.

### 2.7. Oncoplots and Combined Analysis of Other Metrics

The R package maftools was used to aggregate, analyze, annotate, and visualize HCC mutation load data. An oncoplot was generated to display mutations in the high- and low-risk groups and to analyze the disparities in TMB and survival rates between the two groups. TIDE score files were obtained from the http://tide.dfci.harvard.edu, accessed on 2 February 2023 website and downloaded for analysis. Sample TMB and TIDE histograms were created, using SPSS, and the R package ggpubr was used to compare TMB and survival differences between the high- and low-risk groups. Additionally, the ggpubr package was employed to examine potential discrepancies in immune checkpoint blockade responses between the two groups.

### 2.8. Exploring Meaningful Gene Function Pathways

The profiler package in R was employed to conduct GO and KEGG enrichment analyses. A significance level of *p* < 0.05 was set, and a false discovery rate (FDR) below 0.05 was considered statistically significant.

### 2.9. Immunological Infiltration and Drug Screening

The R package limma GSVA was used to analyze immune-related differences in patients with HCC at a significance level of *p* < 0.05. Additionally, therapeutic agents were screened, and drug sensitivity was observed using pRRophetic, ggplot2 and ggpubr with pFilter = 0.001 and corPvalue = 0.001.

### 2.10. Statistical Analysis

The data analysis was performed using R 4.0.3 statistical software. Risk factors were screened using Cox regression analysis. The predictive performance of the model was assessed by evaluating the ROC curve, C-index and calibration curve.

## 3. Results

### 3.1. Clinical Sample Information

The study utilized 424 tissue samples from TCGA, consisting of 375 HCC tissues and 50 normal tissues. Clinical data from 375 patients were retrieved from TCGA, which included information on age, gender, TNM stage, pathological stage, T stage, N stage, M stage, survival time, and survival status. After filtering out missing data, 370 clinical data were obtained, which were matched with the expression data samples (Table 1). The clinical data including gender, age, type of treatment, pathological stage, tumor lymph node metastasis stage, survival time, and previous history of malignant tumor were not statistically different between the two groups (test and train).

### 3.2. Identification of Cuproptosis- and m6A- Related lncRNAs

**Figure 1:**
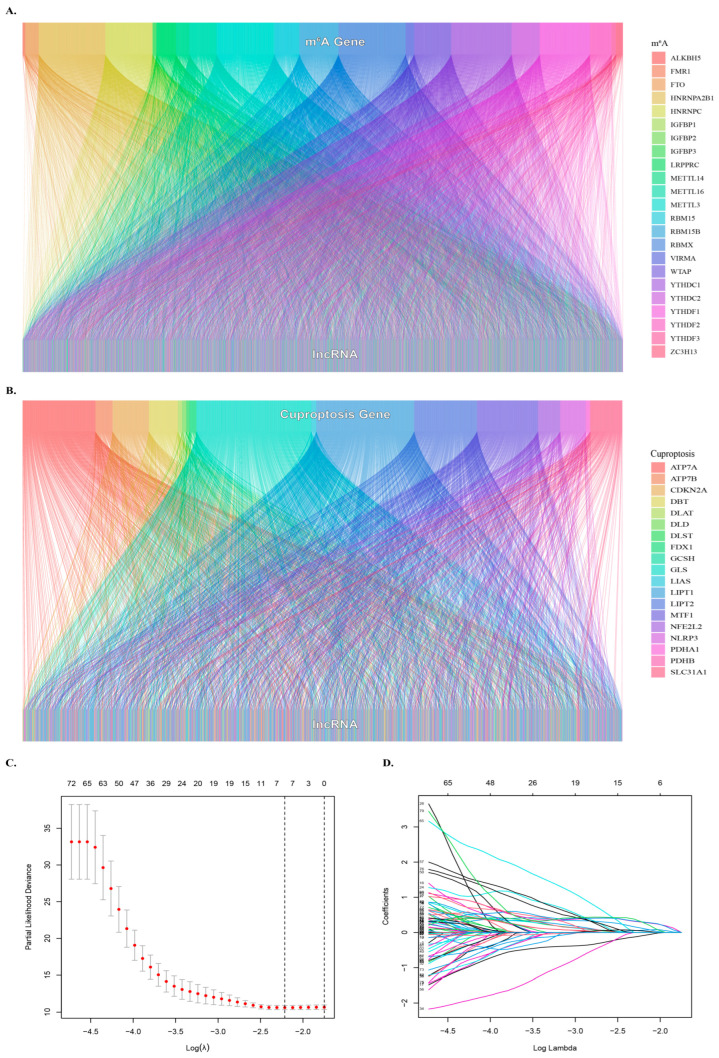
Identification of cuproptosis- and m6A-related lncRNAs. (**A**) Sankey diagram showing the relationship between m6A genes and lncRNAs. (**B**) Sankey diagram showing the relationship between cuproptosis genes and lncRNAs. (**C**) Optimal λ value selection for Lasso regression analysis. (**D**) LASSO regression analyses were used to screen lncRNAs..

In this study, a total of 1348 lncRNAs related to m6A were obtained, and the co-expression network of lncRNA related to m6A was constructed (Figure 1A). Analysis of lncRNAs related to cuproptosis resulted in 1180 lncRNAs related to cuproptosis (Figure 1B). The intersection of the two sets of lncRNAs resulted in 916 common lncRNAs, referred to as mcplncRNAs in consecutive analyses. Univariate Cox regression analysis identified 110 lncRNAs significantly associated with the OS of patients. The forest plot shows the risk profiles of different molecules (Supplementary Figure S1). A total of five mcplncRNAs with independent OS-related risk models were obtained from LASSO survival regression analysis (Figure 1C,D). The risk scores were calculated using:

0.6446 × EXPR(AC007405.3) + 0.6886 × EXPR(AL031985.3) + 1.0170 × EXPR(TMCC1-AS1) + 0.2296 × EXPR(MIR210HG) + 0.5439 × EXPR(TMEM220-AS1).

### 3.3. Prognostic Impact of Mcplncrnas at Different Risks on HCC

**Figure 2:**
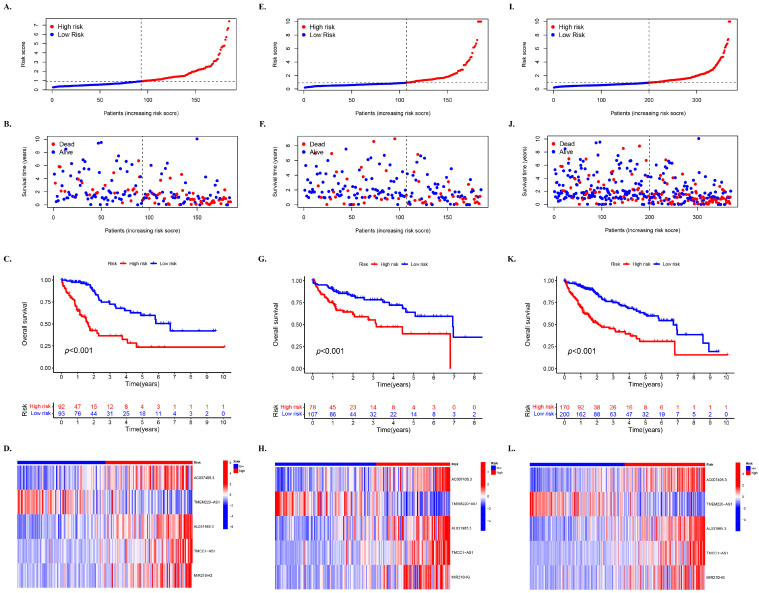
Prognostic impact of mcplncRNAs on HCC for high- and low-risk groups. The distribution of cases in different risk groups based on mcplncRNA model’s risk score in the (**A**) training, (**E**) testing, and (**I**) entire set. The distribution of dead and alive cases in different risk groups based on survival time in the (**B**) training, (**F**) testing, and (**J**) entire set. The distribution of cases in different risk groups based on survival time in (**C**) training, (**G**) testing, and (**L**) entire set. Survival analysis of cases in different risk groups in the (**D**) training, (**H**) testing, and (**L**) entire set.

The training set was used to determine the prognostic risk, where all HCC samples were categorized into high-risk and low-risk groups. Figure 2A demonstrates the distribution of risk levels between the two groups, while Figure 2B shows the survival status and time of patients in both groups. Survival analysis indicated that the OS of the high-risk group was significantly lower than that of the low-risk group (*p* < 0.001) (Figure 2C). The relative expression of the five lncRNAs required for the model in different risk subgroups is depicted in Figure 2D.

Moreover, the distribution of risk levels in the test set (Figure 2E–H) and the entire set (Figure 2I–L) is presented, including the expression of lncRNAs required for the model, as well as survival status and time. Based on the survival analysis results of the test set and the entire set, the outcomes were consistent with those of the TCGA training set. Patients with HCC and higher risk scores had inferior OS compared to those with lower risk scores (Figure 2G,K).

### 3.4. Prognosis Stratified by Various Clinicopathological Features in Different Risk Subgroups

**Figure 3:**
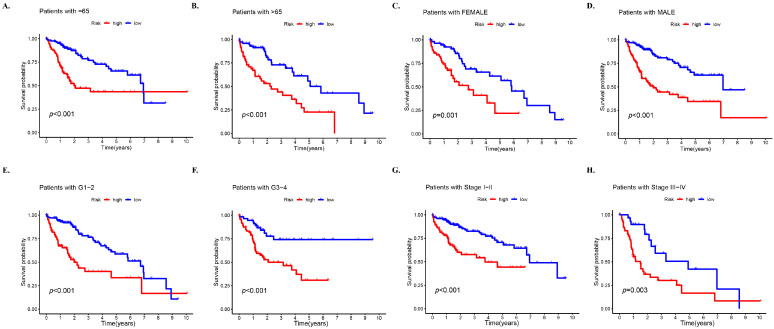
Prognosis stratified by various clinicopathological features in different risk groups. Survival analysis of high- and low-risk groups in the entire set based on (**A**) patients with age of 65 years or less, (**B**) patients with age > 65 years, (**C**) female patients, (**D**) male patients, (**E**) stages G1–2, (**F**) stages G3–4, (**G**) stages I–II, and (**H**) stages III–IV.

Subsequently, an analysis was conducted in the complete dataset to determine differences in OS among different risk subgroups that were stratified by common clinicopathological features. For most low-risk groups, the OS was always higher than that of high-risk groups based on characteristics such as gender and age, which confirmed the prediction accuracy of the model (Figure 3). However, it was observed that patients younger than 65 years of age had better survival in the high-risk group than in the low-risk group after 7 years (Figure 3A) (*p* < 0.01). Moreover, a greater OS in the high-risk group was observed after 9 years in patients at stages G1–G2 (Figure 3E) and about 8.6 years in patients at stages III–IV (Figure 3H).

### 3.5. PCA of Different Risk Groups Based on Selected Factors

**Figure 4:**
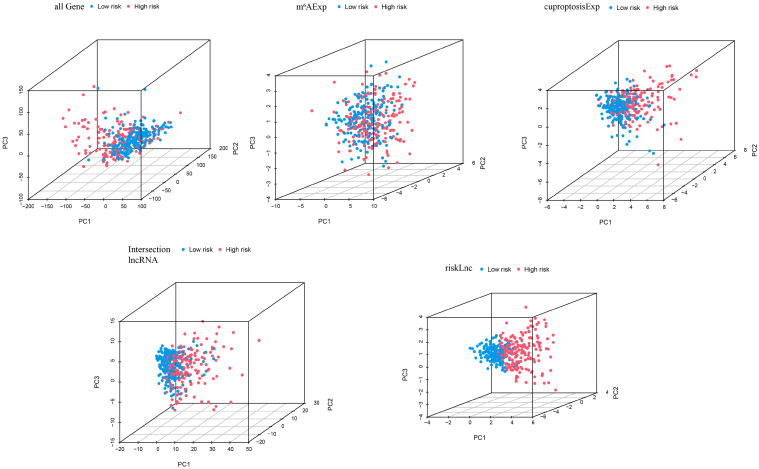
Principal component analysis (PCA) of cases in high- and low-risk groups based on all genes, cuproptosis-related lncRNAs, m6A-related lncRNAs, intersection lncRNAs, and risk model lncRNAs.

The PCA was conducted on all samples based on five dimensions: all genes, cuproptosis-related lncRNAs, m6A-related lncRNAs, intersection lncRNAs, and risk model lncRNAs (Figure 4). The separation between the high- and low-risk groups became more obvious in the same order as mentioned above, indicating that the risk model has a stronger ability to classify the samples.

### 3.6. Multivariate Survival Regression Analyses for Verifying the Prognostic Ability of the Risk Model

**Figure 5:**
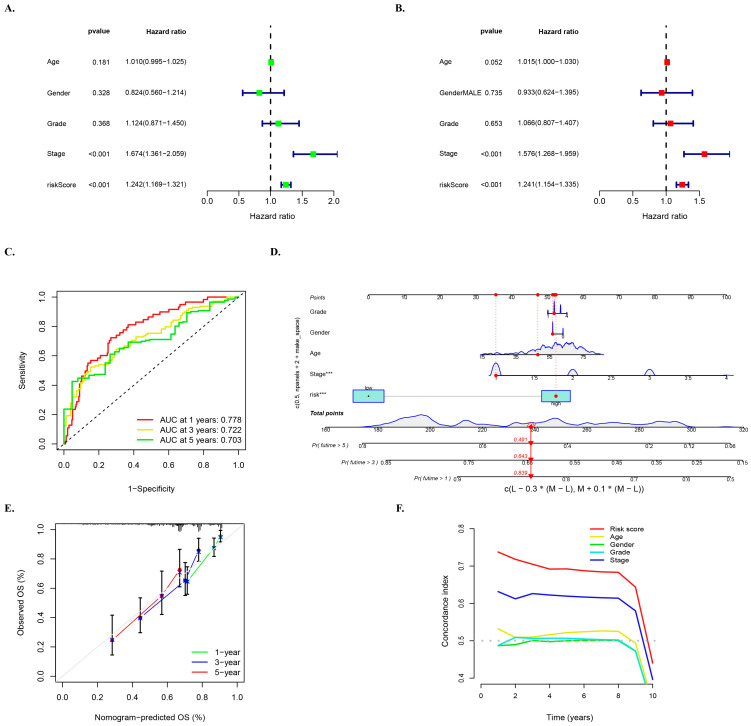
Multivariate survival regression analyses for verifying the prognostic ability of the risk model. (**A**) Univariate and (**B**) multivariate Cox survival regression analyses in the entire cohort for the mcplncRNAs risk score and other clinical characteristics: age, gender, and tumor grade and stage. The notation “***” represents *p* < 0.001. (**C**) ROC curve of the risk model at 1, 3, and 5 years. (**D**) Nomogram for risk score and clinical characteristics. (**E**) Calibration curve of nomogram. (**F**) C-index curve of risk score and clinical characteristics.

Univariate and multivariate survival regression analyses were utilized to investigate the independent prognostic characteristics of HCC risk models of the five required lncRNAs. The univariate Cox regression analysis in the entire cohort revealed a hazard ratio (HR) of 1.242 and 95% confidence interval (CI) of 1.169–1.321 (*p* < 0.001) for the risk score, while the multivariate Cox regression analysis showed an HR of 1.241 with 95% CI of 1.154–1.335 (*p* < 0.001) (Figure 5A,B). To further assess the predictive value of the risk score in HCC prognosis, ROC curves of the model at different times were plotted, and the results indicated that the AUC values were relatively stable at 1 year (0.778), 3 years (0.722), and 5 years (0.703), with little difference observed between the AUC values at different times (Figure 5C). These finding suggest that the five mcplncRNAs have good prognostic risk prediction value for HCC.

Moreover, ROC curves of the model and other clinical characteristics of the entire set of patients at 1 year, 3 years, and 5 years were drawn to better evaluate the ability of the model to distinguish patients and its superiority as an independent prognostic factor under different time conditions (Supplementary Figure S2). These results showed that risk grouping outperformed age, sex, and grading in terms of value.

A nomogram was developed based on multivariate regression analysis of the entire set of HCC patients, which integrated multiple variables such as gender, age, tumor grade and stage, and risk score. Variables in the model showed a close relationship with HCC and could be used to quantitatively predict the survival of HCC patients based on clinical characteristics and scores of the model (Figure 5D). The calibration curve of the nomogram predicted for 1 year, 3 years, and 5 years showed good agreement with the gray dotted line (Figure 5E). Additionally, the C-index curve of the model consistently demonstrated higher C-index value of the model’s rick score than other clinical characteristics (Figure 5F), indicating the reliable prediction accuracy of the model.

### 3.7. TMB and Related Prognosis in High- and Low-Risk Groups

**Figure 6:**
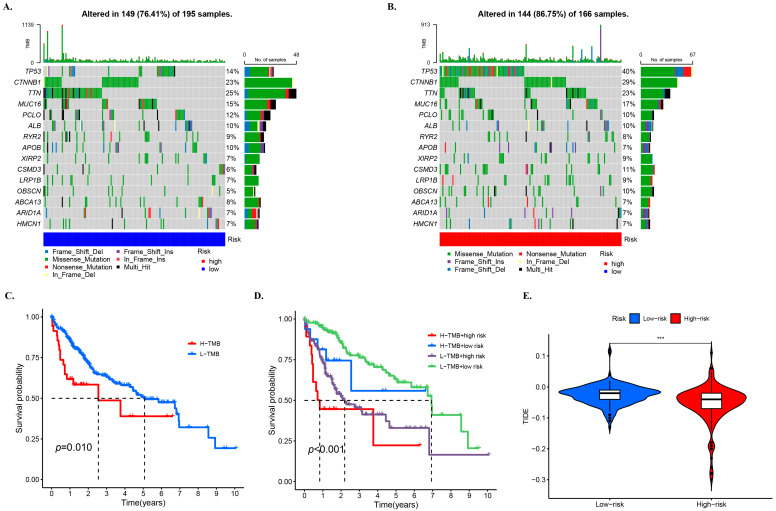
TMB and related prognosis in high- and low-risk groups. SNP alterations identified in (**A**) low- and (**B**) high-risk groups. The KM for subgroups based (**C**) high and low TMB, (**D**) high and low TMB and high- and low-risk groups. (**E**) TIDE scores for the high- and low-risk groups. The notation “***” represents *p* < 0.001.

In this study, waterfall diagrams for the low-risk group (Figure 6A) and high-risk group (Figure 6B) were presented, which showed that the top two genes with mutations in patients were TP53 and CTNNB1. Although we also observed a high frequency of mutations in TTN, and previous studies have reported that its large gene size often leads to false positives [29]. Therefore, we did not focus on the status of this gene in our analysis. Missense mutation, frameshift deletion mutation, and multi-hit mutation were the main mutation types in the low-risk group. Nonsense mutations also accounted for a portion of the proportion in both groups. Based on the median TMB scores, the samples were divided into high and low TMB groups. A survival curve was plotted, which showed that the low TMB group had better survival rate (Figure 6C). Further division of the samples into four groups, i.e., high-risk + high TMB, high-risk + low TMB, low-risk + high TMB, and low-risk + low TMB, revealed that the high-risk + low TMB group had the worst survival rate, while the low-risk + high TMB group had a better survival rate (Figure 6D), which was consistent with the prediction. A violin plot of TIDE between the low-risk and high-risk groups was then drawn, and it was found that the TIDE in the low-risk group was slightly larger than in the high-risk group, which was similar to the projection of the model (Figure 6E).

### 3.8. GO and KEGG Analyses for the Functional Enrichment of Differential Genes in Different Risk Groups

**Figure 7:**
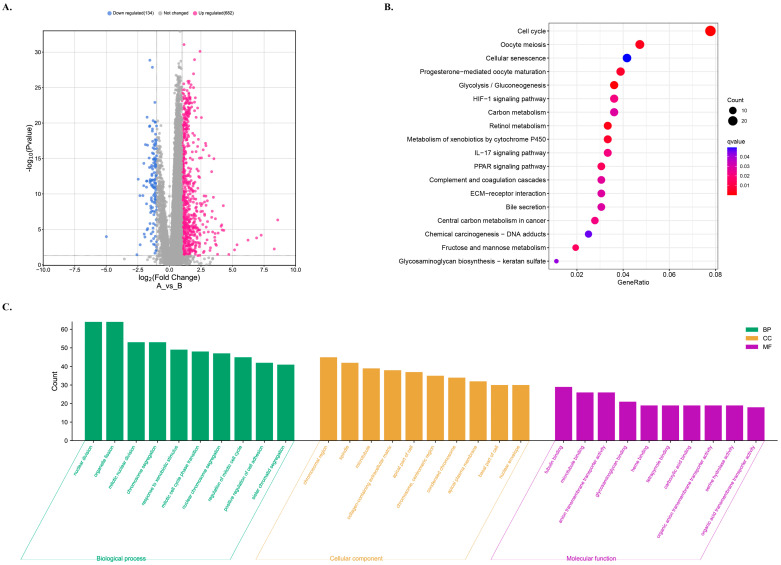
GO and KEGG analyses for the functional enrichment of differentially expressed genes (DEGs) in different risk groups. (**A**) Volcano plot of differentially expressed gene profiles for different risk groups. The low risk as the control. (**B**) KEGG enrichment analysis of the DEGs. (**C**) GO enrichment analysis for DEGs in high-risk group.

The enrichment analyses were performed to obtain information on the diversity of differentially expressed genes (DEGs) in different risk groups, including relevant functions, signaling pathways, and biological processes. The analysis identified a total of 816 DE-lncRNAs, out of which 682 were upregulated and 134 were downregulated in the high-risk groups (Figure 7A).

To understand the functions and action pathways of DEGs, KEGG pathway enrichment analysis was performed. The high-risk group showed changes in the expression of genes mainly involved in the pathways such as cell cycle, oocyte meiosis, cell senescence, progestin-mediated oocyte maturation, and glycolysis/glucose production pathways (Figure 7B). Additionally, KEGG enrichment analysis was used to explore the potential molecular mechanisms based on the model, which revealed immune-related biological processes such as the IL-17 signal pathway (Figure 7B). The roles of the upregulated genes in the high-risk group were investigated in terms of molecular function, biological process, and cellular component GO terms, as shown in Figure 7C.

## 4. Discussion

HCC is a complex disease with multiple factors contributing to its pathogenesis [30]. While exploring the role of specific genes and signaling pathways is important, it is just one aspect of the overall molecular mechanism of HCC [31]. Despite significant improvement in treatment methods, the clinical prognosis for HCC remains suboptimal. Alpha-fetoprotein (AFP) is a well-known biomarker in HCC, but its clinical application is limited by low sensitivity and specificity [32]. Therefore, it is crucial to explore the role of additional molecules in the molecular mechanism of HCC to gain better understanding of the disease and develop precise management. Bioinformatics analysis combined with the construction of multi-gene prediction models is conducive to the development of personalized treatment plans and targeted drugs. In particular, changes in lncRNA expression levels have been linked to various types of cancer and could serve as potential markers for cancer diagnosis and drug targets.

Current research has shown that cuproptosis and m6A play important roles in regulating tumor progression, but their relationship on HCC has not been extensively studied. In this study, we used the TCGA database to identify co-expressed lncRNAs related to 19 cuproptosis-related genes and 23 m6A-related genes. Univariate Cox analysis and LASSO Cox regression analysis were used to identify five lncRNAs (AC007405.3, TMEM220-AS1, AL031985.3, TMCC1-AS1, MIR210HG) with prognostic value in HCC, which have been linked to immune regulation and prognosis of gastric cancer, breast cancer, and liver cancer [33,34,35,36,37]. Our results suggest that investigating the regulatory mechanisms of these molecules may lead to the new targeted therapy and improved prognosis of HCC patients.

The survival analysis demonstrated that the high-risk group had a significantly lower overall survival rate than the low-risk group in both the training and test sets. Patient deaths increased with an increase in risk score, as indicated by the survival status and risk score diagrams, with most samples in the high-risk group experiencing mortality. The ROC curve confirmed the high predictive accuracy of risk score model, while PCA analysis validated the scientific grouping model. Independent prognostic analysis indicated that clinical stage and risk score were independent prognostic factors. The C-index and calibration curve results showed that clinical stage and risk score could accurately predict the survival time of HCC patients. In summary, the risk model based on the five mcplncRNAs performed better than other clinical factors in predicting clinical prognosis, with an increase in risk score being significantly associated with HCC progression. While the relationship between copper and the occurrence and development of HCC is not conclusive, this study insights into the role of cuproptosis- and m6A-related lncRNA in HCC and facilitates the evaluation of HCC prognosis more accurately according to the risk grouping of HCC patients. The discovery provides a new therapeutic target for HCC. Although this finding provides a new target for the treatment of HCC. However, the current data still need to be validated by a larger cohort model and clinical samples. Although we are aware of their importance and our group is actively constructing a larger HCC sample bank and cohort model, we will further validate the expression of related molecules and their application value in large-scale cohort samples in future studies.

## 5. Conclusions

In conclusion, the present study has successfully developed a reliable model of cuproptosis- and m6A-related lncRNAs for the prognosis of HCC. These findings provide new insights into predicting the survival rate of HCC patients and could be useful in determining the efficacy of clinical treatment.

## Figures and Tables

**Table 1:** Characteristic of HCC patients.

Covariates	Type	Total	Test	Train	*p* Value
Age	≤65	232 (62.7%)	116 (62.7%)	116 (62.7%)	1
	>65	138 (37.3%)	69 (37.3%)	69 (37.3%)	
Gender	FEMALE	121 (32.7%)	59 (31.89%)	62 (33.51%)	0.82
	MALE	249 (67.3%)	126 (68.11%)	123 (66.49%)	
Grade	G1	55 (14.86%)	23 (12.43%)	32 (17.3%)	0.37
	G2	177 (47.84%)	92 (49.73%)	85 (45.95%)	
	G3	121 (32.7%)	59 (31.89%)	62 (33.51%)	
	G4	12 (3.24%)	8 (4.32%)	4 (2.16%)	
	unknow	5 (1.35%)	3 (1.62%)	2 (1.08%)	
Stage	Stage I	171 (46.22%)	87 (47.03%)	84 (45.41%)	0.24
	Stage II	85 (22.97%)	49 (26.49%)	36 (19.46%)	
	Stage III	85 (22.97%)	36 (19.46%)	49 (26.49%)	
	Stage IV	5 (1.35%)	2 (1.08%)	3 (1.62%)	
	unknow	24 (6.49%)	11 (5.95%)	13 (7.03%)	
T	T1	181 (48.92%)	91 (49.19%)	90 (48.65%)	0.1
	T2	93 (25.14%)	55 (29.73%)	38 (20.54%)	
	T3	80 (21.62%)	33 (17.84%)	47 (25.41%)	
	T4	13 (3.51%)	5 (2.7%)	8 (4.32%)	
	unknow	3 (0.81%)	1 (0.54%)	2 (1.08%)	
M	M0	266 (71.89%)	129 (69.73%)	137 (74.05%)	1
	M1	4 (1.08%)	2 (1.08%)	2 (1.08%)	
	unknow	100 (27.03%)	54 (29.19%)	46 (24.86%)	
N	N0	252 (68.11%)	121 (65.41%)	131 (70.81%)	0.57
	N1	4 (1.08%)	3 (1.62%)	1 (0.54%)	
	unknow	114 (30.81%)	61 (32.97%)	53 (28.65%)	

T: Tumor, N: Node, M: Metastasis.

## Data Availability

Data available on request.

## Supplementary Materials

The following supporting information can be downloaded at: https://www.mdpi.com/article/10.3390/biology12081101/s1, Supplementary Figure S1: Univariate survival analysis forest map; Supplementary Figure S2: ROC curves for age, grade, and risk at one, three and five years.
