# Supplementary figures and images for: Cuproptosis- and m6A-Related lncRNAs for Prognosis of Hepatocellular Carcinoma
Source: Biology (Basel). 2023 Aug 8;12(8):1101. doi: 10.3390/biology12081101 (PMC10451969; doi:10.3390/biology12081101)

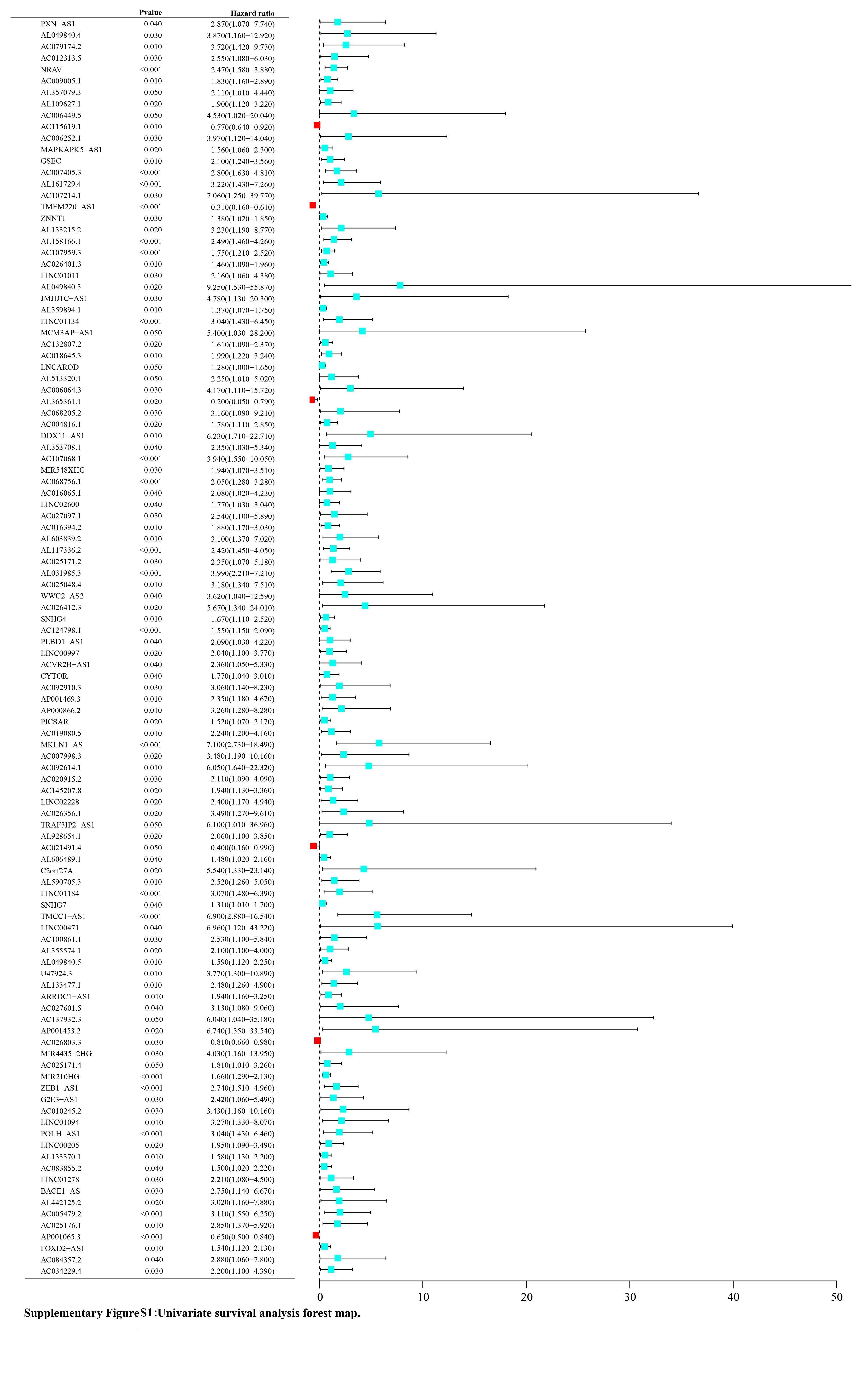

Supplement: Supplementary file 1 [file biology-12-01101-s001.zip › Supplementary Figure S1.jpg]

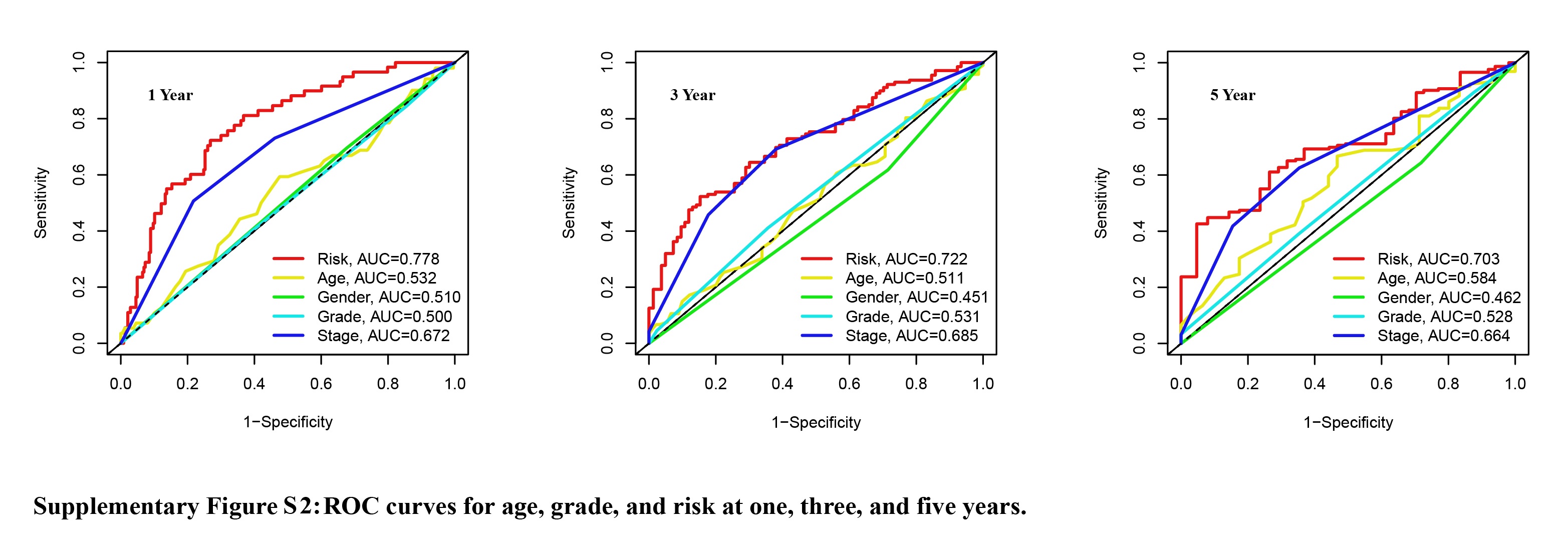

Supplement: Supplementary file 1 [file biology-12-01101-s001.zip › Supplementary Figure S2.jpg]
